# Diffuse Idiopathic Pulmonary Neuroendocrine Cell Hyperplasia (DIPNECH): A Case of Indolent Pulmonary Nodules Diagnosed with Robotic-Assisted Navigational Bronchoscopy

**DOI:** 10.1155/2021/6312296

**Published:** 2021-12-10

**Authors:** Adam Purdy, Firas Ido, Deborah Stahlnecker

**Affiliations:** Division of Pulmonary and Critical Care, St. Luke's University Health Network, Bethlehem, PA, USA

## Abstract

Diffuse idiopathic pulmonary neuroendocrine cell hyperplasia (DIPNECH) is an atypical pulmonary disorder with limited understanding. Given the rare nature of this disease, it is essential to obtain adequate tissue pathology to confirm the diagnosis. This disease is mainly diagnosed in middle-aged, nonsmoking females, and it is now accepted as a precursor lesion to pulmonary carcinoid tumors. DIPNECH presents with characteristic radiographic and histologic findings, but its diagnosis, management, and prognosis are often underrecognized and poorly understood. Those with symptoms may present with shortness of breath, wheezing, and persistent cough and are often misdiagnosed with reactive airway disease. Pulmonary function testing may reveal airflow obstruction and air trapping. Imaging is characterized by multiple lung nodules, typically less than 5 mm in size, with a background mosaic attenuation on computed tomography imaging. Histologically, DIPNECH can be suspected based on the presence of hyperplastic neuroendocrine cells. DIPNECH is considered a precursor to invasive neuroendocrine tumor, and up to 50% of patients may have a well-differentiated neuroendocrine tumor at the time of presentation. Here, we present the case of a 46-year-old female with a history of ulcerative colitis on mesalamine who presented with a 6-month history of ongoing shortness of breath, chest tightness, wheezing, and cough. She was initially diagnosed with asthma before imaging later revealed as multiple pulmonary nodules with a diffuse mosaic pattern. Using robotic-assisted navigational bronchoscopy, she underwent sampling of a dominant 1.8 cm right middle lobe pulmonary nodule and pathology was consistent with low-grade neuroendocrine tumor.

## 1. Introduction

Diffuse idiopathic pulmonary neuroendocrine cell hyperplasia (DIPNECH) is a rare pulmonary disorder that is being reported with increasing frequency. It was not fully recognized and reported until 1992, when it was described in 6 nonsmoking, mainly female patients, who presented with cough and shortness of breath (1). DIPNECH is now recognized by the World Health Organization (WHO) classification of lung tumors as a preneoplastic lesion (2). Surgical lung biopsy is accepted as the “gold standard” for diagnosis. However, navigational bronchoscopy has emerged as a less-invasive and important modality to aid in the diagnosis of this rare disease.

## 2. Case

**Figure 1:**
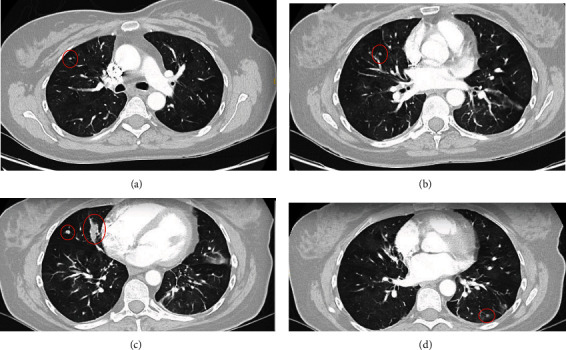
CT chest with diffuse mosaic attenuation and multiple pulmonary nodules: (a) 4 mm right upper lobe nodule; (b) 4 mm right middle lobe nodule; (c) 7 mm lateral right middle lobe nodule with confluent 1.8 cm medial right middle lobe nodule; (d) 4 mm left lower lobe nodule.

**Figure 2:**
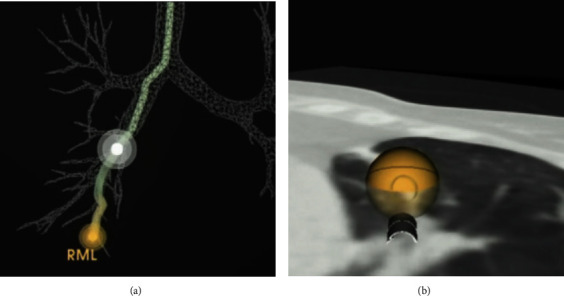
(a) Monarch robotic-assisted navigational bronchoscopy path to the right middle lobe lung nodule. (b) Axial computed tomography imaging superimposed on the robotic-assisted pathway to the right middle lobe lung nodule.

**Figure 3:**
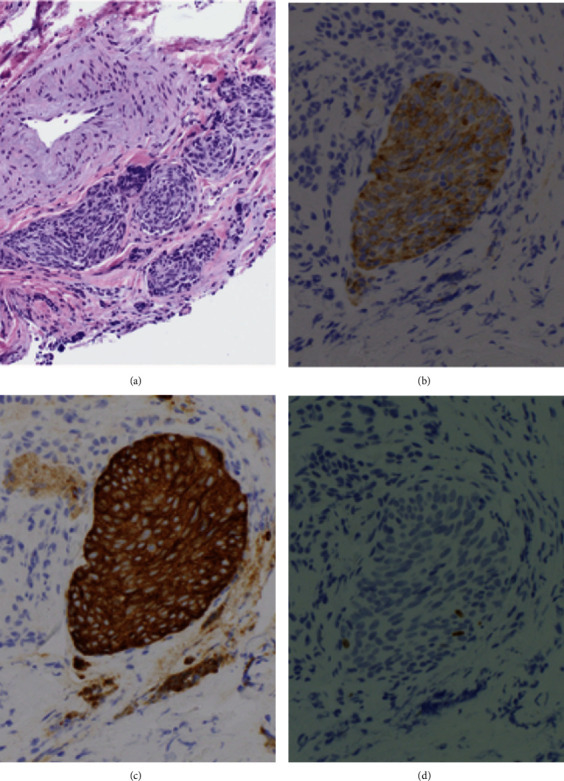
(a) H&E stain of the medial right middle lobe nodule. (b) Medial right middle node nodule with chromogranin-positive pathology, a neuroendocrine marker. (c) Medial right middle lobe nodule with synaptophysin-positive pathology, another marker for neuroendocrine cells. (d) Medial right middle lobe nodule with a Ki-67 proliferation marker with only 3 nuclei staining positive.

We present the case of a 46 year-old-female, never smoker, with a history of ulcerative colitis who initially presented as an outpatient for the evaluation of persistent cough, wheezing, and chest tightness for 6 months. Her review of systems was otherwise negative and her only medication included mesalamine for ulcerative colitis. She had no history of environmental or occupational exposures and denied any allergies. She denied any personal or family history of lung disease. Her vital signs were within normal limits. This patient was evaluated by pulmonology and was diagnosed with cough variant asthma and was started on montelukast and albuterol as needed. A northeast allergy panel was unremarkable. She subsequently presented to the emergency room with an episode of wheezing and chest tightness and Computed Tomography Angiogram (CTA) revealed multiple lung nodules with mosaic attenuation. The nodules were seen bilaterally in clusters, with the largest measuring up to 1.8 cm in the right middle lobe. Multiple serologic markers were ordered to screen for autoimmune disease including ANA, ANCA, anti-dsDNA, hypersensitivity pneumonitis panel, Sjogren's antibodies, angiotensin converting enzyme, and rheumatoid factor. These were unremarkable other than a positive ANA with titer 1 : 640 homogenous pattern. Due to uncontrolled symptoms, her inhaler regime was escalated to Flovent twice daily with as needed albuterol. A repeat CT chest 3 months later indicated no change in diffuse mosaicism and multiple pulmonary nodules, similar in size, with the largest 1.8 cm in the right middle lobe (Figures 1(a)–1(d)). She then underwent robotic-assisted navigational bronchoscopy with fine needle aspiration, brushing, and transbronchial biopsy of the right middle lobe nodule (Figures 2(a) and2(b)). Pathology from the nodule was positive for groups of bland appearing small blue cells, consistent with low-grade neuroendocrine tumor (carcinoid). Pathology was positive for chromogranin and synaptophysin, neuroendocrine markers, and low Ki-67 positivity, indicating low rate of proliferation (Figures 3(a)–3(d)). Endobronchial ultrasound was also performed and Level 7 lymph node was negative for malignancy. The patient was presented at multidisciplinary chest tumor conference and it was recommended that she undergo PET CT scan and surgical resection of the biopsy-proven typical carcinoid tumor in the right middle lobe.

## 3. Discussion

Diffuse idiopathic pulmonary neuroendocrine cell hyperplasia (DIPNECH) is a rare pulmonary condition. This condition leads to a proliferation of neuroendocrine cells that can be confined to the lung mucosa, cause local invasion, or progress into invasive carcinoid tumors. It is primarily diagnosed in middle-aged, nonsmokers, with a female predominance (3).

Most symptomatic patients present with a long history of cough, shortness of breath, and wheezing. These patients are often misdiagnosed with asthma and there is a significant time lag before the correct diagnosis is made. The average length of time between symptom onset and diagnosis is between 9 and 16 years (4). Most patients have airflow obstruction and air trapping on pulmonary function testing. Radiographic imaging may reveal nodular bronchial wall thickening caused by intraluminal protrusion of proliferating cells, bilateral pulmonary nodules (typically 5 mm or less), ground glass mosaicism consistent with air trapping, and bronchiectasis (3). Pulmonary nodules are assumed to be well-differentiated lung neuroendocrine tumors and most express somatostatin receptors (5). Histologically, the cells are round or spindle-shaped and have a moderate amount of eosinophilic cytoplasm with round to oval nuclei. Constrictive obliterative bronchiolitis, which represents the histological hallmark of DIPNECH, is indicated by mild, chronic inflammatory cell infiltrate and wall thickening and fibrosis of involved airways, leading to narrowing and possible obliteration of the bronchial lumen (2). Due to the presence of neuroendocrine tumorlets and hyperplasia, DIPNECH is considered a precursor to invasive neuroendocrine tumor and, at diagnosis, approximately 50% of patients have a synchronous well-differentiated neuroendocrine tumor (6). Given this, tumors greater than 5 mm in size can be considered carcinoid tumors and should be biopsied (7). It is essential to obtain adequate pathology and bronchoscopy with navigation may be needed for peripheral nodules. Expression of somatostatin receptors in tumorlets and carcinoid cells can be demonstrated by scintigraphy with octreotide or histology (8).

Due to the rare nature of this disease, data on treatment and outcomes are limited. The majority of patients are treated with steroid-based therapy (inhaled and systemic), in an effort to reduce the inflammatory response induced by neuroendocrine cell-secreted neuropeptides (9). Bronchodilator therapy can be beneficial in treating symptoms. Octreotide, a somatostatin analogue, has been used with some success in the presence of somatostatin receptors. This is similarly used to reduce the hormonal hypersecretion of neuroendocrine cells in gastrointestinal and bronchial carcinoids (10). The course of this disease is extremely variable. Many patients will have a slowly progressive or stable clinical course, while fewer may have marked constrictive bronchiolitis, which may progress to severe airflow obstruction.

## 4. Conclusion

There are no well-established guidelines for the diagnosis, treatment, and management of diffuse idiopathic pulmonary neuroendocrine cell hyperplasia (DIPNECH), given the rarity of this disease. It should not be overlooked given its classification as a preneoplastic process and ability to transform into invasive carcinoid tumor. Our patient was found to have a low-grade carcinoid tumor, and it was recommended that she undergo resection. Given the rarity of DIPNECH, long-term prognosis is variable and this patient must be monitored closely.
